# Calcifying Epithelial Odontogenic Tumor

**DOI:** 10.1155/2013/725380

**Published:** 2013-11-27

**Authors:** Olavo Hoston Gonçalves Pereira, Laura Priscila Barboza de Carvalho, Vilson Lacerda Brasileiro Junior, Cláudia Roberta Leite Vieira de Figueiredo

**Affiliations:** ^1^Saint Vincent de Paul Hospital, João Pessoa, PB, Brazil; ^2^UNIPE, João Pessoa, PB, Brazil; ^3^Department of Odontology, School of Dentistry, University Center of João Pessoa (UNIPE), BR- 230, Km 22, Água Fria, 58053-000 João Pessoa, PB, Brazil; ^4^Federal University of Rio Grande do Norte (UFRN), Natal, RU, Brazil; ^5^UFPB, João Pessoa, PB, Brazil; ^6^University of São Paulo (USP), Brazil

## Abstract

The calcifying epithelial odontogenic tumor (CEOT) is a rare benign epithelial odontogenic neoplasm of slow growth that is locally aggressive and tends to invade bone and adjacent soft tissue. Here is reported the case of a 21-year-old female patient with a CEOT in the left mandibular posterior region. The computerized tomography in coronal plane revealed a hypodense lesion in the posterior region of the left mandibular body with hyperdense areas inside and was associated with element 37. An incisional biopsy of the lesion was performed and the histopathological analysis revealed the presence of layers of epithelial odontogenic cells that formed prominent intercellular bridges. A large quantity of extracellular, eosinophilic, and amyloid-like material and an occasional formation of concentric calcifications (Liesegang rings) were also found. The histopathological diagnosis was a Pindborg tumor. Resection of the tumor with a safety margin was performed and after 6 months of follow-up there has been no sign of recurrence of the lesion.

## 1. Introduction

The calcifying epithelial odontogenic tumor (CEOT) or Pindborg tumor is a rare benign epithelial odontogenic neoplasm [1, 2], with approximately 200 reported cases in the literature [3]. In general it occurs as a slow-growing tumor [4], associated with an impacted tooth in the posterior mandibular region [5, 6]. This occurs predominantly between the fourth and fifth decades of life, with no preference of gender [2–4]. Radiographically, CEOT is characterized by a uni- or multilocular lesion that often shows a mixed radiolucent-radiopaque pattern [6]. Treatment consists in the surgical removal of the lesion, with recurrence in 14% of cases [5]. The prognosis is considered good [6].

## 2. Case Report

**Figure 1:**
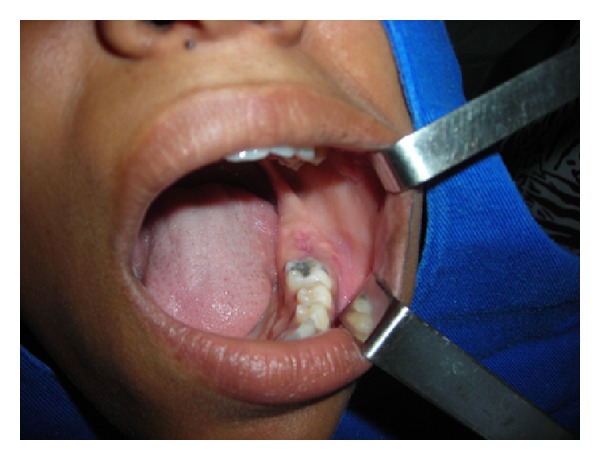
Intraoral aspect of the lesion, characterized by a hardened nodular and exophytic mass in the mandibular body, on the left side.

A 21-year-old black female patient sought treatment at the Oral and Maxillofacial Trauma Surgery Unit, with swelling in the mandibular left posterior region. While performing the intraoral physical exam an asymptomatic hardened exophytic nodule was found in region 37 (Figure 1).

**Figure 2:**
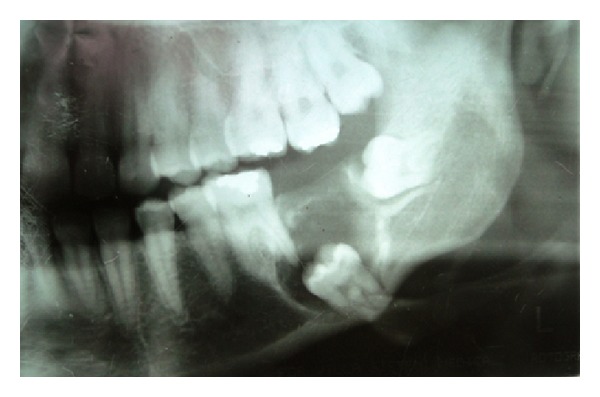
A panoramic radiograph showing a unilocular, radiolucent lesion, diffuse, associated with dental element 37.

**Figure 3:**
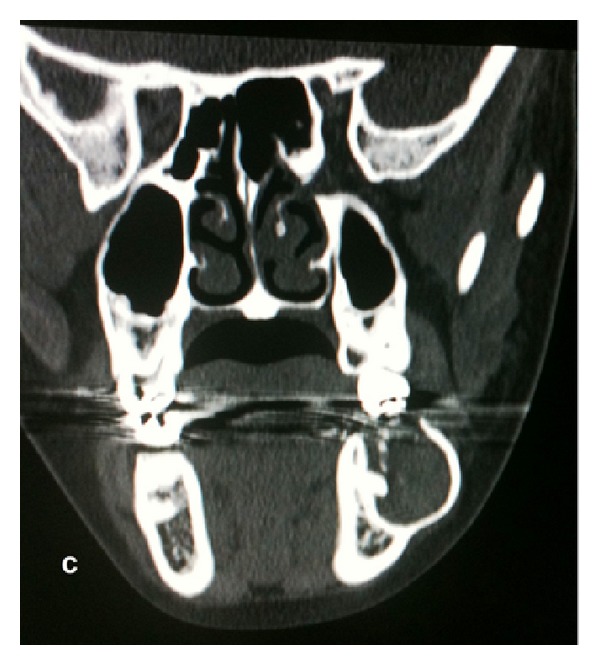
Coronal CT showing a hypodense lesion, containing irregular hyperdense areas in its inside, associated with the expansion of the vestibular cortical bone and the resorption of the cortical bone of the alveolar process.

The patient reported a clinical evolution of only five months of the lesion. A radiographic exam revealed a diffuse radiolucent, unilocular lesion, associated with region 37, yet including the distal root of region 36 up to the crown of region 38 (Figure 2). Using a computerized tomography in coronal section, it was possible to observe that it was a question of a local hypodense lesion in the mandibular region, containing irregular hyperdense areas in its midst, compatible with the presence of mineralized tissue in the lesion. Further, it was also noted that the tumor caused expansion of the vestibular cortical bone and showed resorption of the cortical bone of the alveolar process (Figure 3). Based on clinical and radiographic findings, diagnostic hypotheses suggested a dentigerous cyst, unicystic ameloblastoma, and CEOT.

**Figure 4:**
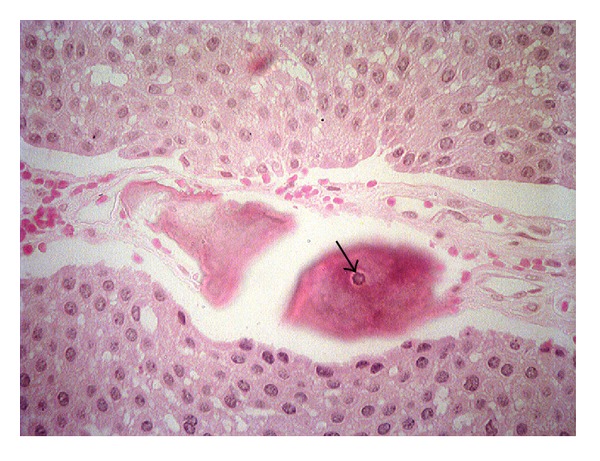
Layers of epithelial odontogenic cells forming prominent intercellular bridges. Areas of extracellular, amyloid-like material, and concentric calcifications (arrow), Liesegang rings (HE/40x).

An incisional biopsy ensued and the specimen was sent to the Laboratory of Oral Pathology. The histopathological analysis revealed layers of odontogenic epithelial cells that formed prominent intercellular bridges. Areas of extracellular, eosinophilic, and amyloid-like material with an occasional formation of concentric calcifications (Liesegang rings) were also present (Figure 4). Based on histological findings, the final diagnosis was a calcification epithelial odontogenic tumor.

Surgical resection of the tumor was performed, including a marginal portion of apparently healthy bone, while attaching a 2.4 mm reconstruction plate of titanium, in search of the patient's rehabilitation. After 6 months of follow-up there has been no sign of recurrence of the lesion.

## 3. Discussion

CEOT is an uncommon lesion which generally affects the posterior maxillary region. A frequent radiographic finding in these tumors has been the presence of calcifying structures of varying sizes inside the lesions. Besides this, the cases reported in the literature have mostly been painless, of a slow evolution, and in an intraosseous area [6–9]. Supporting the literature, the present study reports the case of a painless tumor, located centrally in the left posterior region of the mandible and showing the presence of calcifying structures in the radiographic exam. However, some of the characteristics found in this case are uncommon when compared with the literature, such as the fact that the tumor has affected a patient below the age of 30 and showed rapid evolution in the mandibular region.

The histological criteria of the Pindborg tumor are layers of polyhedral epithelial cells with well-defined borders that oftentimes show prominent intercellular bridges. Figures of mitosis are rarely seen. In the layer of epithelial cells, circles, full of a homogenous amyloid-like substance, were observed. Some of those cells were also filled with a calcifying matter in the form of Liesegang rings, which are pathognomonic of this tumor [3–5]. The histopathological examination of this reported case showed clear characteristics of CEOT like the presence of amyloid-like matter, the concentric rings of Liesegang, and the presence of prominent intercellular bridges.

Generally its treatment is undergone beginning with the surgical excision of the tumor. As the lesion was not encapsulated, the majority of authors agree that resection should include a safety margin of bone that is considered clinically and radiographically healthy [5, 10]. The Pindborg tumor is a rare lesion, so there are no large series of patients with follow-up [10]. Consequently the treatment undergone and the recurrence factor are arguable, although the majority of authors agree that 1 cm safety margin is sufficient. In the present case, after 6 months of follow-up, there was no clinical evidence of recurrence of the lesion. However, the patient will be periodically examined over the next five years to verify the possible recurrence of the lesion.
